# Risk Factors, Lifestyle and Prevention among Adolescents with Idiopathic Juvenile Scoliosis: A Cross Sectional Study in Eleven First-Grade Secondary Schools of Palermo Province, Italy

**DOI:** 10.3390/ijerph182312335

**Published:** 2021-11-24

**Authors:** Dalila Scaturro, Claudio Costantino, Pietro Terrana, Fabio Vitagliani, Vincenzo Falco, Daniele Cuntrera, Claudia Emilia Sannasardo, Francesco Vitale, Giulia Letizia Mauro

**Affiliations:** 1 Department of Surgery, Oncology and Stomatology, University of Palermo, 90127 Palermo, Italy; dalila.scaturro@unipa.it (D.S.); terranapietro@gmail.com (P.T.); giulia.letiziamauro@unipa.it (G.L.M.); 2 Department of Health Promotion, Maternal Infant Care, Internal Medicine and Excellence Specialties, “G. D’Alessandro”—University of Palermo, 90127 Palermo, Italy; claudiaemilia.sannasardo@unipa.it (C.E.S.); francesco.vitale@unipa.it (F.V.); 3 Faculty of Medicine and Surgery, University of Catania, 95100 Catania, Italy; fabiovitagliani93@libero.it; 4 Department of Statistics, University of Palermo, 90127 Palermo, Italy; vincenzo.falco01@unipa.it; 5 Department of Economics and Statistics, University of Palermo, 90121 Palermo, Italy; daniele.cuntrera@unipa.it

**Keywords:** idiopathic adolescent scoliosis, risk factors, first-grade secondary school student, back pain, high risk sport, dysmorphism of the developmental age, preventive strategies

## Abstract

Adolescent idiopathic scoliosis (AIS) has an incidence of 2–3% in the general population and a multifactorial etiology. The present study aims to analyze modifiable risk factors and their interactions in the development of AIS in order to increase knowledge about the disease and to prevent the evolution of AIS in young students with tailored public health strategies. A cross-sectional study was conducted over two consecutive school years among students attending 11 first-grade secondary schools in the province of Palermo, Italy. A self-administered questionnaire that investigated socio-demographical, physical and anamnestic characteristics and habits, focusing on possible risk factors associated with idiopathic scoliosis, was administered. In addition, a clinical evaluation was performed with Adams’ test and Bunnel’s inclinometer. Suspected AIS cases were associated with the practice of high-risk sports (*p* < 0.05), weekly physical activity lasting ≥3 h (*p* < 0.05), lower back pain (*p* < 0.001), posture disorders (*p* < 0.01) and having had no contact with a physician (*p* < 0.01). Practice of high-risk sports (adj OR = 1.83; CI 95% 1.11–4.76) and suffering of posture disorders (adj OR = 1.67; CI 95% 1.12–3.60) showed a significant association with a confirmed diagnosis of AIS (Cobb angle ≥ 10° at X-ray). The risk factors associated with AIS are still unclear. Therefore, it is crucial to identify early modifiable and multiple risk factors to prevent the evolution of scoliosis in school-age children.

## 1. Introduction

Adolescent idiopathic scoliosis (AIS) is a three-dimensional deformity of the spine in the three planes of space [1,2,3]. Its prevalence is between 0.47% and 5.2%, with an incidence of the disease equal to 2–3% in the general population and a female/male ratio equal to 4:1 [4].

Generally, in adolescents, the deformation of the spine is mild (10° Cobb angle); in two-thirds of cases, the curve reaches a 20–30° Cobb angle, and in 10% of cases, the curve reaches 40° (severe scoliosis) [5].

Scoliosis can be congenital, but in most cases, it is an acquired disease. In about 90% of cases, the acquired form does not recognize a precise cause and it is classified as idiopathic [6].

To date, there is a standard agreement that scoliosis has a multifactorial etiology. Genetic predisposition certainly plays a crucial role in scoliosis development; in fact, monozygotic twins have a high concordance rate of idiopathic scoliosis [7]. On the other hand, recent studies have supported the polygenic origin of scoliosis, resulting from the interaction between multiple gene loci and the environment [5].

Various possible risk factors related to the onset of scoliosis have been studied in the literature, such as growth alterations, postural disorders, heavy backpacks, environmental factors, high-risk sports and visual and dental disorders [8].

To date, a clear relationship has not yet been identified between scoliosis and the adoption of poor postures or carrying heavy backpacks [9,10]. Likely, the adoption of inappropriate sitting posture for long periods or excessive weight of backpacks could lead to fatigue of the paravertebral muscles and an increase in pressure on the ligaments, predisposing one to the development of scoliosis. However, there are not enough data to support this relationship [9,10].

The onset of scoliosis could be related to sports activity. It has been shown that high-risk sports practices, such as ballet, rhythmic gymnastics, swimming and other athletic activities, can increase the risk of developing a deformity of the column [6].

Furthermore, a possible relationship between strabismus and scoliosis has recently been highlighted. Patients with strabismus show a much higher risk of developing dorsal scoliosis, as they can present an altered visual–spatial perception with asymmetrical postural responses [11].

Nevertheless, there is no certain relationship between one or more risk factors and the insurgence of AIS.

The objective of the present study was to clarify the possible role of modifiable risk factors associated with juvenile idiopathic scoliosis in order to increase knowledge of the disease and to suggest tailored preventive strategies for high-risk groups, such as the large-scale organization of screening campaigns and educational interventions on risk factors for adolescents directly at school.

## 2. Materials and Methods

### 2.1. Study Design

A multidisciplinary cross-sectional study coordinated by the Rehabilitation and Health Promotion Departments at the University of Palermo, Italy, was conducted over two consecutive school years (2018/2019 and 2019/2020) in 11 first-grade secondary schools located in the province of Palermo. An informative note on the objectives and purposes of the study was provided to all parents of the students attending the schools involved in the study, and a consent/dissent form was completed in order to agree to the participation to the survey and to the educational interventions on adolescent idiopathic scoliosis.

The survey was carried out with a pre-intervention questionnaire, administered in paper form, before moving onto an educational intervention for adolescent idiopathic scoliosis. 

A self-administered questionnaire that investigated socio-demographical, physical and anamnestic characteristics and habits, focusing on possible risk factors associated with idiopathic scoliosis, was previously validated in a preliminary pilot testing study conducted among 30 students of first-grade secondary schools during the month of October 2018. 

The reliability and validity of the questionnaire were evaluated and Cronbach’s alpha was calculated as 0.86, demonstrating adequate reliability of the test. 

The pre-intervention questionnaire included 29 items and was divided into three sections:

1. The first section included 9 questions regarding socio-demographic and general characteristics of pre-adolescents (age, sex, height, weight, number of family members, parents’ educational level, parents’ occupation);
2. The second section consisted of 8 questions concerning the daily personal habit and attitudes of the study sample (physical activity duration and type, use of backpack and daily meters traveled, daily time spent sitting, personal smartphone or tablet and daily usage); and
3. The third section included 12 items regarding clinical characteristic, attitudes and habits associated with scoliosis of the adolescents (visual, hearing, dental or posture disorders and type, back pain and daily duration, familiarity with scoliosis, talking about scoliosis with a physician).

An educational intervention on adolescent idiopathic scoliosis on associated risk factors and on protective factors to avoid scoliosis, conducted by Public Health and Physiatrists medical residents at the University of Palermo, was carried out. Supporting the intervention, a set of slides related to adolescent idiopathic scoliosis was presented in plenary sessions to all students. All students underwent a specialist psychiatric examination and were evaluated through the Adams test and the Bunnel inclinometer. 

In order to compare the survey with the specialist medical visit, all participants were requested to generate a unique anonymous code at the beginning. The unique code was formed by the initials of the research center, the initial of the name and surname and the birth month of the student. Any adolescent found positive to one of the three screening tests successively underwent at the rehabilitation medical unit of the University Hospital of Palermo a radiographic exam of the column, under load, in two projections (A/P and L/L), as a further instrumental diagnostic investigation, also with measurements of Cobb’s Angle and Risser grade. 

The study was approved by the Ethical Committee Palermo of the University Hospital of Palermo (5/2019 of 22 May 2019) with the frame of rules specified by the Declaration of Helsinki and its subsequent amendments, and the principles of good clinical practice.

### 2.2. Statistical Analysis

Data collected through the questionnaire were included in a database created with EpiInfo 7.3.2.1 (Centers for Disease Control and Prevention, Atlanta, GA, USA). 

All data were analyzed using the statistical software package Stata/MP 12.1 (StataCorp LP, College Station, TX, USA).

Absolute and relative frequencies were calculated for the categorical (qualitative) variables, and quantitative variables were summarized by their means (standard deviations).

The differences in the categorical variables for a suspected diagnosis of scoliosis with socio-demographic, daily personal habits/attitudes and clinical characteristics were analyzed in a univariate analysis using chi-squared tests (Mantel–Haenszel). 

A *p*-value less than 0.05 was considered significant in this model.

To guarantee a more conservative approach, all socio-demographic, personal habits/attitudes and clinical variables found to have an association with a confirmed diagnosis of scoliosis (Cobb angle ≥ 10° at X-ray) in the univariate analysis with a *p*-value ≤ 0.20 were included in a multivariate logistic regression model. 

The crude odds ratio (crude OR) and the adjusted OR (adj-OR) with 95% confidence intervals (Cis) were calculated in the logistic regression model. The level of significance chosen for the multivariate logistic regression analysis was 0.05 (two-tailed).

## 3. Results

**Figure 1:**
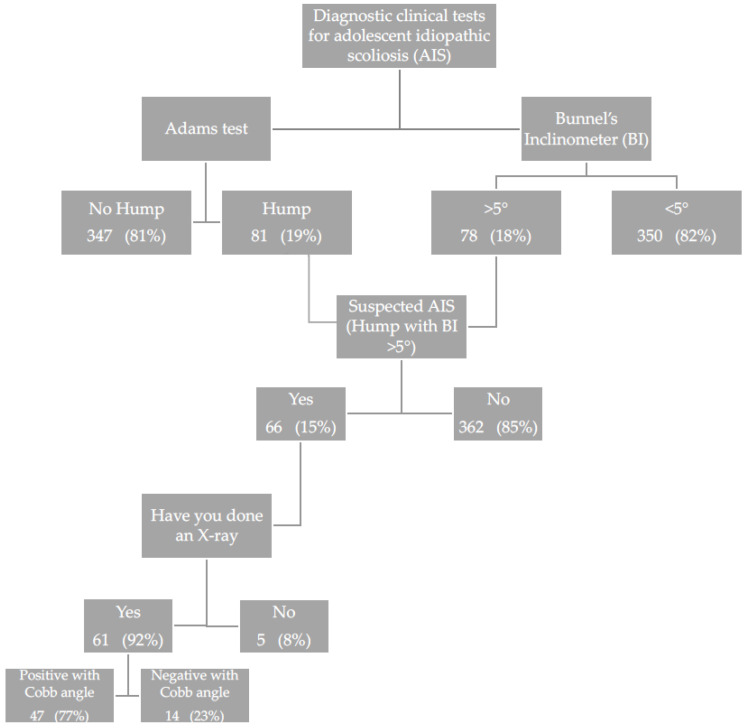
Flow chart of diagnostic clinical tests specific for scoliosis in the study sample (*n* = 428).

In Figure 1 are reported diagnostic clinical tests specific for scoliosis offered after the intervention to the adolescents. In 19% of adolescents, Adam’s test was positive. Bunnel’s inclinometer is considered positive when the angle of rotation is >5°, seen in 18% of respondents.

A total of 428 students attending first-grade secondary schools of Palermo province aged between 11 and 14 years old (mean age = 11.7 years) were enrolled in the study.

In the study, the suspicion of adolescent idiopathic scoliosis was confirmed when Adam’s test was positive and was associated with Bunnel’s inclinometer >5.

In total, 15.4% of the participants met the criteria of suspicion for adolescent idiopathic scoliosis and 92.4% of them underwent a radiographic exam of the column, under load, in two projections (A/P and L/L), as further instrumental diagnostic investigation that confirmed the diagnosis suspicion.

In the end, 47 participants were confirmed with the diagnosis of scoliosis by measuring Cobb angle during an X-ray.

Table 1 shows the results of the univariate analysis about the correlation of suspected adolescent idiopathic scoliosis in relation with socio-demographic factors (gender, height, weight, BMI), with daily personal habit and attitudes of pre-adolescents enrolled in the first-grade secondary schools of the Palermo district. 

In particular, a total of 43 students with suspicion of adolescent idiopathic scoliosis practice physical activity and play high-risk sports for scoliosis. For respondents with suspicion of scoliosis who practice sports, the duration of physical activity for 41 of them was less than three hours per week.

A significant association with suspected adolescent idiopathic scoliosis among pre-adolescents of first-grade secondary schools enrolled in the study was reported between students who practiced high risk sport such as dance or artistic gymnastics (*p*-value = 0.04) and who generally practiced physical activity less than 3 h weekly (*p*-value = 0.03).

On the other hand, a significant association was not found for the use of the backpack, the daily meters traveled with a backpack and the time spent sitting daily.

In Table 2, the results of the univariate analysis about the correlation of suspected adolescent idiopathic scoliosis in relation with clinical characteristic, attitudes and habits among all of participants in the sample are reported.

Most of the sample correlated to the suspected adolescent idiopathic scoliosis did not suffer from back pain (51 vs. 15 subjects).

Among adolescents with suspected idiopathic scoliosis, 12 students had posture disorders and 16 students had familiarity with scoliosis.

Moreover, in this table, the daily duration of back pain (more than 3 h a day, *p*-value < 0.001) and diagnosed posture disorders (*p*-value < 0.01) were also statistically significantly associated with suspected adolescent idiopathic scoliosis.

Finally, talking about the risk of scoliosis with a physician was associated with suspected adolescent idiopathic scoliosis (*p*-value < 0.01).

Finally, in Table 3, crude ORs and adjORs of variables (socio-demographic, daily personal habit, attitudes, clinical characteristics of students) associated with confirmed diagnosis of scoliosis (Cobb angle ≥ 10° at X-ray) are reported.

The practice of high risk sports (adj OR = 1.83; CI 95% 1.11–4.76) and suffering of posture disorders (adj OR = 1.67; CI 95% 1.12–3.60) showed a significant association with confirmed diagnosis of scoliosis (Cobb angle ≥ 10° at X-ray) at the multivariable analysis.

## 4. Discussion

The multifactorial etiology of scoliosis is now well known. However, the knowledge regarding the possible risk factors associated with juvenile idiopathic scoliosis is still unclear. Therefore, it is of crucial importance to identify the modifiable risk factors that can influence the disease and to define appropriate strategies aimed at preventing them.

One of the most important secondary preventive tool that allows us to carry out adequate prevention strategies is screening, especially in the groups most at risk (i.e., in the case of AIS, the 11–14 years age group) [12,13,14].

Screening for the early diagnosis of deformity of the spine in adolescents is most effective if performed at regular intervals during the main growth stages [15].

Implementing a prevention program based on an educational intervention that increases knowledge of the disease is desirable, not only for adolescents who fall into high-risk categories. In this regard, the drafting of an easy-to-perform questionnaire that requires very little time to implement can be a useful tool in identifying the physical characteristics and personal habits that predispose adolescents to scoliosis development.

The collaboration between the National Education System (and specifically among schools) and the National Health System (NHS) could allow the early detection of paramorphism and dimorphisms in adolescence throughout the organization of tailored screening programs for school-aged adolescents. During this period of life, acting on the risk factors determining scoliosis becomes of fundamental importance to reduce its evolution and take on real meaning in terms of prevention. In the conservative treatment of scoliosis, a late diagnosis is often a risk of survival, i.e., warranting the need for surgery.

Sport can be synonymous with “harmonic” prevention activities, such as Pilates, which can improve the expansion of the rib cage and the flexibility of the trunk [16].

Other sports such as football, basketball, etc., by improving neuromotor control, provide support to the trunk and pelvis, all conditions capable of preventing spinal deformities [17].

On the other hand, it is not possible to identify the sports that place participants at risk for this pathology; however, it is necessary to not subject the spine to excessive asymmetries or mobilizations, as in dance and rhythmic gymnastics, because this would make it more easily deformable and favor vertebral rotation.

To confirm this, in our experience, it emerged that practicing a sport such as rhythmic gymnastics or dance and/or presenting other paramorphism predisposes adolescents to the onset of scoliosis, so much so that they can be considered “red flags” and require a diagnostic check. In accordance with our data, Watanabe et al. [7] observed an increased likelihood of developing scoliosis in girls who practiced classical ballet.

The authors also noted an increase in the likelihood of scoliosis with increasing frequency and duration of classical ballet training. Conversely, what happens in sports such as basketball and volleyball appears to be associated with a jump in the likelihood of drains [7].

Previous studies have also shown a high correlation between developmental dysmorphism and scoliosis [18,19]. Therefore, children in whom developmental dimorphism is identified should be considered at high risk of developing scoliosis and should be followed closely over time.

Even the number of hours dedicated to sport per week would appear to influence the risk of developing the disease; in fact, at least three hours of physical activity per week are recommended.

Additionally, most of the students examined used smartphones/tablets and carried heavy backpacks.

There may be a possible relationship between these factors and scoliosis, but this association has not been demonstrated. This is in line with the data present in the literature; in previous studies, Minghelli et al. [9] tried to investigate the role of heavy backpacks in influencing the onset of scoliosis without showing a relationship between the two factors.

Finally, we studied the etiological and dental role in the onset of scoliosis as they can lead to proprioceptive dysregulation and impact the maintenance of a correct posture [11].

However, from our experience, although a large part of the sample studied suffered from visual and/or dental disturbances, there was no association with the onset of scoliosis.

Another element to underline is that the presence of lower back pain in adolescents should be investigated and considered as a wake-up call. The presence of back pain is a fairly common symptom in adolescents with scoliosis and deserves special clinical attention [20].

Back pain in AIS has different causative factors and could also be associated with psychological and morphological parameters [21]. For instance, pain catastrophizing or lumboischialgia were also significant factors in the onset of back pain and should be taken into consideration when evaluating these patients [21,22].

There are some limitations of the present study that need to be highlighted. Firstly, a possible lack of representativeness due to the limited number of participants should be considered. Nevertheless, the large majority of students who attended the 11 first-grade secondary schools participated to the study (>98%) and the distribution of schools throughout the province of Palermo guaranteed the enrollment in the study sample of students with different sociodemographic variables.

Another limit of our study derives from its transversal nature, which does not allow us to evaluate a cause and effect relationship, but only an association between various risk factors and the presence of musculoskeletal disorders through a multivariable analysis.

In the present study, we have shown that, among adolescents with scoliosis, the development of lower back pain compared to the same healthy population is twice as likely.

Therefore, the presence of lower back pain should be investigated and considered as an alarm bell.

Raising awareness, educating and anticipating possible risk behaviors are the only real prevention methods that, together with early diagnosis, are effective.

Screening has proven to be an effective tool to identify patients at risk or suffering from juvenile idiopathic scoliosis without any waste of resources, without the possibility of complications and without the execution of unnecessary specialist visits. A greater diffusion of this screening method and better school information campaigns would be desirable.

## 5. Conclusions

The present study showed that suffering from back pain for more than 3 h a day, having developmental dysmorphism and playing a high-risk sport (such as gymnastics) increases the risk of developing scoliosis among adolescents. The study’s strengths are represented by the originality of the work, as it simultaneously evaluates multiple risk variables and the formulation of an easily reproducible questionnaire. However, further cross-sectional studies on a larger sample are needed in the future to confirm and strengthen the data presented.

## Figures and Tables

**Table 1:** Sociodemographic characteristics and daily personal habits of pre-adolescents enrolled in the first-grade secondary schools of the Palermo district of the study sample and association in the univariate analysis to suspected adolescent idiopathic scoliosis (*n* = 428).

Variables		Yes *n* = 66, *n* (%)	No *n* = 362, *n* (%)	*p*-Value
Age in years (mean)	- Mean ± SD	11.74 ± 0.83	11.77 ± 0.85	0.84
Gender	- Male	32 (14)	196 (86)	0.23
	- Female	34 (17)	166 (83)	
Height	- ≤140 cm	10 (13.5)	64 (86.5)	0.3
	- >140 cm	56 (15.8)	298 (84.2)	
Weight	- ≤40 kg	23 (13.7)	145 (86.3)	0.25
	- >40 kg	43 (16.5)	217 (83.5)	
BMI (kg/m^2^)	- Underweight	24 (12.9)	162 (87.1)	0.17
	- Healthy weight	38 (16.6)	191 (83.4)	
	- Overweight/Obese	4 (30.8)	9 (69.2)	
Physical activity	- Yes	43 (17)	209 (83)	0.16
	- No	23 (13)	153 (87)	
If yes, it is a high risk sport (dance or artistic gymnastics)?	- Yes	11 (28)	28 (72)	0.04
	- No	32 (15)	181 (85)	
Weekly duration of physical activity	- ≤3 h	41 (18.9)	176 (81.1)	0.03
	- >3 h	2 (5.7)	33 (94.3)	
Use of backpack	- Yes	64 (16)	337 (84)	0.18
	- No (use bag with wheels)	2 (7.4)	25 (92.6)	
Daily meters traveled with a backpack	- <300 mt	52 (15.2)	292 (84.8)	0.41
	- >300 mt	14 (16.7)	70 (83.3)	
Daily time spent sitting	- <8 h	47 (15)	266 (85)	0.40
	- >8 h	19 (16.5)	96 (83.5)	

**Table 2:** Clinical characteristics, attitudes and habits of pre-adolescents enrolled in first-grade secondary schools of the Palermo district of the study sample and association in the univariate analysis to suspected adolescent idiopathic scoliosis (*n* = 428).

Variables		Yes *n* = 66, *n* (%)	No *n* = 362, *n* (%)	*p*-Value
Visual disorders	- Yes	28 (16.8)	139 (83.2)	0.31
	- No	38 (14.5)	223 (85.5)	
If yes, what kind of disorders	- Astigmatism/hypermetropia	8 (12.3)	57 (87.7)	0.15
	- Myopia	20 (19.6)	82 (80.4)	
Dental disorders	- Yes	14 (22.2)	49 (77.8)	0.08
	- No	52 (14.2)	313 (85.8)	
If yes, what kind of disorders	- Dental malocclusion	5 (38.5)	8 (61.5)	0.11
	- Dental device	9 (18)	41 (82)	
Back pain	- Yes	15 (14)	92 (86)	0.38
	- No	51 (15.9)	270 (84.1)	
Daily duration of back pain	- <3 h	9 (9.4)	87 (90.6)	<0.001
	- >3 h	6 (54.5)	5 (45.5)	
Posture disorders	- Yes	12 (34.3)	23 (65.7)	<0.01
	- No	54 (13.7)	339 (86.3)	
If yes, what kind of disorders	- Deformity of the spinal column	4 (36.4)	7 (63.6)	0.8
	- Height difference of the basin	6 (40)	9 (60)	
	- Difference in shoulder blades	2 (22.2)	7 (77.8)	
Familiarity with scoliosis	- Yes	16 (18.4)	71 (81.6)	0.24
	- No	50 (14.7)	291 (85.3)	
Talking about scoliosis with physician	- Yes	22 (23.6)	71 (76.3)	<0.01
	- No	44 (13.2)	290 (86.8)	

**Table 3:** Crude ORs and adjORs of variables (socio-demographic, daily personal habit, attitudes, clinical characteristics of students) associated with confirmed diagnosis of scoliosis (Cobb angle ≥ 10° at X-ray).

Variables	Crude OR	CI 95%	*p*-Value	AdjOR	CI 95%	*p*-Value
**Gender**						
- Male	ref		0.21			
- Female	1.47	(0.80–2.70)				
**Age in years (continuous variable)**	1.09	(0.77 1.55)	0.61			
**BMI (kg/m^2^)**						
- underweight	ref		0.09	ref		0.07
- overweight/healthy weight	1.60	(0.92–2.68)		1.68	(0.97–2.95)	
**Physical activity**						
- Yes	ref		0.91			
- No	1.03	(0.55–1.91)				
**Physical activity (high risk sports)**						
- No	ref		<0.05	ref		<0.05
- Yes	1.78	(1.06–5.06)		1.83	(1.11–4.76)	
**Weekly duration of physical activity**						
- ≤3 h	ref		0.28			
- >3 h	0.44	(0.1–1.96)				
**Daily meters traveled with a backpack**						
- <300 mt	ref		0.49			
- >300 mt	1.29	(0.63–2.65)				
**Daily time spent sitting**						
- <8 h	ref		0.83			
- >8 h	0.92	(0.46–1.85)				
**Visual disorder**						
- No	ref		0.40			
- Yes	1.30	(0.70–2.40)				
**Dental disorders**						
- No	ref		0.18	ref		0.49
- Yes	1.67	(0.78–3.56)		0.99	(0.34–2.80)	
**Back pain**						
- No	ref		0.79			
- Yes	0.90	(0.44–1.85)				
**Posture disorders**						
- No	ref		<0.05	ref		<0.05
- Yes	2.69	(1.14–6.23)		1.67	(1.12–3.60)	
**Familiarity with scoliosis**						
- No	ref		0.35			
- Yes	1.39	(0.69–2.32)				
**Talking about scoliosis with physician**						
- No	ref		0.08	ref		0.24
- Yes	1.81	(0.93–3.51)		1.55	(0.74–3.23)	

## Data Availability

The datasets generated during and/or analyzed during the current study are available from the corresponding author on reasonable request.
